# CCCTC-Binding Factor Acts as a Heterochromatin Barrier on Herpes Simplex Viral Latent Chromatin and Contributes to Poised Latent Infection

**DOI:** 10.1128/mBio.02372-17

**Published:** 2018-02-06

**Authors:** Jennifer S. Lee, Priya Raja, Dongli Pan, Jean M. Pesola, Donald M. Coen, David M. Knipe

**Affiliations:** a Department of Microbiology and Immunobiology, Harvard Medical School, Boston, Massachusetts, USA; b Program in Virology, Harvard Medical School, Boston, Massachusetts, USA; c Department of Biological Chemistry and Molecular Pharmacology, Harvard Medical School, Boston, Massachusetts, USA; University of Michigan—Ann Arbor

**Keywords:** chromatin, epigenetics, herpes simplex virus, latent infection, regulation of gene expression

## Abstract

Herpes simplex virus 1 (HSV-1) establishes latent infection in neurons via a variety of epigenetic mechanisms that silence its genome. The cellular CCCTC-binding factor (CTCF) functions as a mediator of transcriptional control and chromatin organization and has binding sites in the HSV-1 genome. We constructed an HSV-1 deletion mutant that lacked a pair of CTCF-binding sites (*CTRL2*) within the latency-associated transcript (*LAT*) coding sequences and found that loss of these CTCF-binding sites did not alter lytic replication or levels of establishment of latent infection, but their deletion reduced the ability of the virus to reactivate from latent infection. We also observed increased heterochromatin modifications on viral chromatin over the *LAT* promoter and intron. We therefore propose that CTCF binding at the *CTRL2* sites acts as a chromatin insulator to keep viral chromatin in a form that is poised for reactivation, a state which we call poised latency.

## IMPORTANCE

Herpes simplex virus 1 (HSV-1) is a human pathogen that persists for the lifetime of the host as a result of its ability to establish latent infection within sensory neurons. The mechanism by which HSV-1 transitions from the lytic to latent infection program is largely unknown; however, HSV-1 is able to coopt cellular silencing mechanisms to facilitate the suppression of lytic gene expression. Here, we demonstrate that the cellular CCCTC-binding factor (CTCF)-binding site within the latency associated transcript (*LAT*) region is critical for the maintenance of a specific local chromatin structure. Additionally, loss of CTCF binding has detrimental effects on the ability to reactivate from latent infection. These results argue that CTCF plays a critical role in epigenetic regulation of viral gene expression to establish and/or maintain a form of latent infection that can reactivate efficiently.

## INTRODUCTION

Herpes simplex virus 1 (HSV-1) undergoes a lytic infection cycle at the primary mucosal site of infection, expresses approximately 80 lytic genes, and then spreads to sensory neurons, where it establishes a latent infection and expresses a minimal number of viral genes (1). Viral gene products recruit host epigenetic complexes to regulate the viral genome during lytic and latent infection (2, 3). HSV-1 persists as a latent infection in sensory ganglia, during which lytic genes are epigenetically silenced, and the only viral gene products expressed abundantly are a family of noncoding RNAs known as the latency-associated transcripts (LATs) and microRNAs (miRNAs) (4–8). The *LAT* gene is transcribed to yield a primary 8.3-kb transcript from which stable 1.5- and 2.0-kb introns and a number of miRNAs are processed (6–9). The LATs promote gene silencing and increased heterochromatin at lytic genes and have been associated with a reduction in lytic gene transcripts in both acute and latent infections of neurons (10–14). In addition, *LAT*-dependent gene repression during latent infection in a mouse model has been implicated in promoting neuronal survival and suppressing reactivation (15). No strong evidence for proteins encoded by the *LAT* genes has been found (16); however, long noncoding RNAs in other systems are known to mediate assembly of heterochromatin and maintenance through both direct and indirect mechanisms (16). In transfection assays, miRNAs originating from the *LAT* region can also repress expression of viral lytic proteins (6, 17–19).

During establishment of latency, lytic gene expression and viral replication are repressed and the viral genome progressively accumulates histones and heterochromatin on lytic genes (20, 21). Viral lytic genes are associated with histones that are hypoacetylated and enriched for markers of heterochromatin, such as histone H3 lysine 9 trimethyl (H3K9me3), which is a hallmark of constitutive heterochromatin, and H3 lysine 27 trimethyl (H3K27me3), which is a hallmark of facultative heterochromatin (9, 20–23). Elements of the *LAT* transcriptional unit, which include upstream regulatory sequences, a neuron-specific promoter, and a downstream enhancer, appear to be the exception to this chromatin phenotype (22–28). The *LAT* gene is the only viral region known to be enriched for acetylated histones and other markers of active euchromatin, while also maintaining association with markers of heterochromatin (22, 23). Upon reactivation *in vitro*, this pattern is reversed, with lytic genes associating increasingly with acetylated histones and markers of euchromatin and accumulating transcripts, while the *LAT* gene exhibits a corresponding decrease in euchromatin and transcript levels (29–32). Chromatin control of viral lytic gene expression is therefore thought to act as a regulator of the transitions between lytic infection, latent infection, and reactivation.

**FIG 1:**
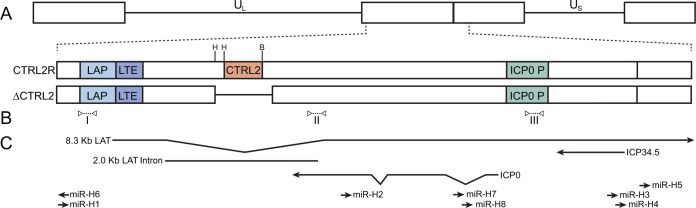
Map of the *LAT* transcriptional unit of an HSV-1 viral mutant with a deletion of the CTCF-binding sites from the *LAT* intron sequences. (A) Schematic map of the HSV-1 genome with an expanded view showing the *LAT* coding region, with the *LAT* promoter (*LAP*), *LAT* enhancer (*LTE*), CTCF-binding sites (*CTRL2*), and *ICP0* promoter (ICP0 P). Restriction endonuclease cleavage sites (H, HpaI; B, BamHI) used to generate the ΔCTRL2 virus are indicated. (B) The locations of qPCR primers are indicated as open arrows connected by dashed lines: I, LAP; II, LAT intron; III, ICP0 P. (C) Locations of the primary *LAT*, stable 2.0-kbp *LAT* intron, *ICP0* transcript, and miRNAs.

Transcribed from the strand antisense to the *LAT* gene in each of the long component repeats is the *ICP0* gene (Fig. 1), which encodes an immediate-early (IE) protein that serves many functions to promote lytic infection, including the transactivation of viral genes and repression of the innate immune system and intrinsic resistance (33–39). ICP0 counters host-mediated chromatin silencing, intrinsic resistance, and innate immune responses through several mechanisms, including the degradation of promyelocytic leukemia (PML) protein in nuclear domain 10 (ND10) bodies (40), degradation of interferon-inducible protein 16 (IFI16) (36), and inhibition of the histone deacetylase (HDAC) RE1-silencing transcription factor (REST) corepressor to REST (CoREST)-HDAC repressor complex (41, 42). In latently infected neuronal populations, *ICP0* is largely repressed despite its proximity to the *LAT* enhancer sequences and abundant expression from the adjacent *LAT* region (43); however, we recently found that ICP0 promotes *LAT* expression and latent infection (44).

The host factors regulating latent viral chromatin have not been well defined. One candidate, CCCTC-binding factor (CTCF), is an 11 zinc-finger DNA-binding protein that is essential, ubiquitously expressed, and highly conserved among metazoan species (45, 46). CTCF was initially classified as a transcriptional repressor with the ability to bind diverse DNA target sequences (45–48) and was later recognized as a transcriptional activator as well as an enhancer blocker and insulator (49–51). Consequently, CTCF emerged as an important chromatin regulator responsible for a diverse range of activities, including affecting pausing of RNA polymerase II (RNAPII) and RNA splicing, directing the specific positioning and phasing of nucleosomes (52, 53), and generating chromatin loops and mediating long-range chromatin interactions through manipulation of the chromatin three-dimensional (3D) architecture (54–56).

Importantly, CTCF is the only identified vertebrate insulator protein. As such, it acts as a boundary element to isolate adjacent domains of active and inactive chromatin by directing enhancer activity to prevent interaction with nearby but inappropriate promoters and by blocking the linear spread of heterochromatin (51, 57–59). Insulators are crucial in the spatial organization of complex genomes of both metazoan species and herpesviruses (60–63), throughout which essential active regions of transcription are frequently interspersed with silenced domains.

CTCF-binding sites have been identified in a number of herpesviruses and are associated with functions such as regulating latent gene expression in two gammaherpesviruses, Epstein-Barr virus (EBV) (60) and Kaposi’s sarcoma-associated herpesvirus (KSHV) (61, 62), as well as regulating expression of the major immediate-early gene in the betaherpesvirus human cytomegalovirus (HCMV) (63). A number of CTCF-binding sites have been identified in the HSV-1 genome, including the *CTRL2* sites, which are located between the *LAT* and *ICP0* promoter regions in the terminal repeats of the long component (Fig. 1) (64, 65). CTCF binds at the *CTRL2* sites during latent infection but is lost during reactivation (66), and this site has been hypothesized to regulate distinct expression from the adjacent genetic elements (64–66). Additionally, in transfection assays, *CTRL2* is capable of enhancer blocking, silencing, and prevention of heterochromatin spreading (64, 65). Indirect evidence also suggests that removal of CTCF-binding sites can alter gene expression from lytic promoters in cell culture latency models (67). Interestingly, in lytic infection, CTCF is not detected at *CTRL2* or other latency-associated binding sites; however, CTCF does bind extensively to other regions of the viral genome and may promote viral transcription and prevent epigenetic silencing (68).

We were therefore interested in determining whether the presence of CTCF binding at the *LAT* intron is critical for establishing and/or maintaining the latent gene expression pattern and chromatin structure in the *LAT* region during latency. In this study, we eliminated the *CTRL2* sites from HSV-1 to assess CTCF function in a mouse model system of latency. We found that the *CTRL2* sites are essential for maintaining the chromatin at the *LAT* and *ICP0* regions that promotes efficient reactivation.

## RESULTS

### Construction of an HSV-1 *CTRL2* binding site deletion mutant virus.

Previous studies identified the *CTRL2* DNA elements within the HSV-1 genome and demonstrated that *CTRL2* was able to bind CTCF both *in vitro* and *in vivo* (64–66) and function as an insulator capable of blocking enhancer-promoter interactions (64) and the spread of heterochromatin in transfection assays (65). To test whether the *CTRL2* region has specific effects on *in vivo* HSV-1 infection, we constructed a *CTRL2* deletion mutant virus by removing a 370-bp fragment from within the 2.0-kbp *LAT* intron to generate the HSV-1 ΔCTRL2 mutant virus (Fig. 1). A control virus, called CTRL2R, was constructed in parallel by restoring an intact *LAT-CTRL2* region in KOSΔLAT1.8eGFP virus, the parent of ΔCTRL2 mutant virus.

**FIG 2:**
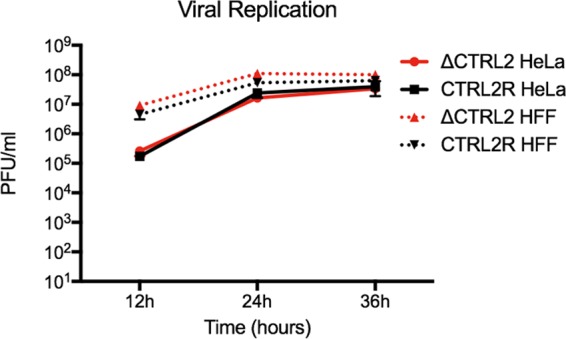
Analysis of ΔCTRL2 and CTRL2R lytic viral replication. HeLa or HFF cells were infected at 0.1 PFU/cell with ΔCTRL2 or CTRL2R virus. Progeny virus was collected at 12, 24, and 36 hours postinfection and titrated on Vero cells.

Growth kinetics in human foreskin fibroblast (HFF) and HeLa cells following infection at a low multiplicity of infection (0.1) confirmed that there were no differences in lytic replication between the ΔCTRL2 mutant and control CTRL2R viruses (Fig. 2).

### Analysis of CTCF binding to the ΔCTRL2 mutant and rescued viral genomes.

**FIG 3:**
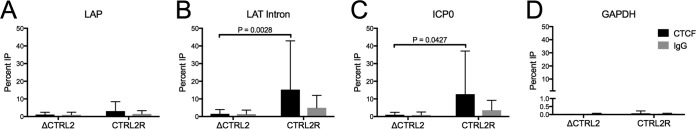
Comparison of CTCF association at the *LAT* region during latent infection with ΔCTRL2 or CTRL2R virus in mice. Mice were infected with ΔCTRL2 or CTRL2R virus. At 28 dpi, the mice were sacrificed, and ChIP experiments were carried out on harvested TGs by using antibodies specific for CTCF. Three viral regions were queried: *LAP* (A), the *LAT* intron (B), and the *ICP0* promoter (C). The cellular *GAPDH* sequences (D) were also analyzed. Percentages of immunoprecipitated DNA are shown as means and standard deviations from 7 ChIP experiments from 4 independent infections. Statistical significance was evaluated using Friedman’s test along with Dunn’s posttests for ΔCTRL2 versus CTRL2R (controlling for multiple comparisons); significant *P* values are shown on top of the brackets.

To confirm the loss of CTCF binding during *in vivo* infection due to deletion of the *CTRL2* site and to validate the mutant virus, we infected mice via corneal scarification with 2 × 10^5^ PFU/eye of ΔCTRL2 or CTRL2R virus and performed chromatin immunoprecipitation (ChIP) for CTCF. At 28 days postinfection (dpi), trigeminal ganglia (TGs) were removed and processed for ChIP analysis with a CTCF-specific antibody, and we quantified the immunoprecipitated DNA sequences by using quantitative PCR (qPCR) with primers specific to the *LAT* promoter, *LAT* intron, and *ICP0* promoter (Fig. 3). ChIP analysis performed in parallel with a nonspecific rabbit IgG confirmed specificity. Statistical analyses were performed on results from 7 independent ChIP experiments from 4 independent infections, using Friedman’s test with Dunn’s *post hoc* test to account for multiple comparisons. Consistent with the absence of putative CTCF-binding sites, CTCF was not enriched at the *LAT* promoter (*LAP*) or at the cellular *GAPDH* pseudogene relative to the nonspecific antibody control upon infection with either ΔCTRL2 or CTRL2R virus (Fig. 3A and D). Comparison of the ΔCTRL2 mutant viral chromatin to control CTRL2R viral genome showed a significant decrease in CTCF binding at the *LAT* intron based on ChIP using primers specific to a region less than 500 bp downstream of the *CTRL2* site (*P* ≤ 0.003) (Fig. 3B). Interestingly, ΔCTRL2 viral genomes also showed a significant reduction of CTCF binding within the *ICP0* promoter region (*P* ≤ 0.05) (Fig. 3C). CTCF enrichment at the downstream *ICP0* promoter site was consistent with a potential CTCF-binding site within the *ICP0* promoter region or detection of CTCF binding to another CTCF-binding site, such as the CTa′_*m*_ site, which is ~1.5 kbp away from *CTRL2* (64). Collectively, these results demonstrated that deletion of the *CTRL2* sequences in the ΔCTRL2 virus reduced CTCF association at the *LAT* intron sequences and at the *ICP0* promoter region relative to that with the control CTRL2R virus.

### Acute infection of mice with ΔCTRL2 and CTRL2R viruses.

**FIG 4:**
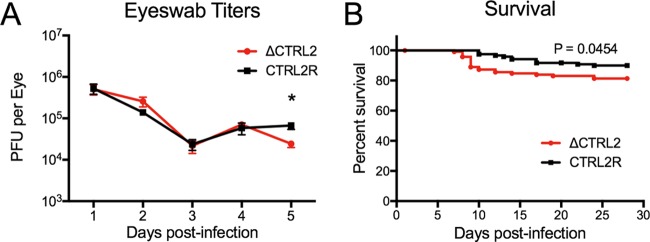
Eye swab titers and survival of mice infected with ΔCTRL2 or CTRL2R virus. Mice were infected with 2 × 10^5^ PFU/eye of ΔCTRL2 or CTRL2R virus. (A) Viral replication in corneal epithelia. Eye swabs were collected daily at 1 to 5 dpi from 5 mice infected with each virus, from 4 independent infections. Collected virus was titrated on Vero cells, and the results are plotted as mean and standard errors for all infections. Statistical significance was evaluated using Student’s *t* test and is indicated with an asterisk (*P* < 0.05). (B) Infections were allowed to progress for 28 days, and survival curves are presented from 8 independent infections of groups of 5 to 20 mice per virus (infected in parallel in each infection group). Statistical significance was evaluated with the log-rank Mantel-Cox test.

We first examined acute infection in mice infected with the ΔCTRL2 and CTRL2R viruses by collecting shed virus with eye swabs for the initial 5 dpi and titrating the virus on Vero cells. We observed similar levels of viral shedding from mice infected with ΔCTRL2 and CTRL2R viruses at days 1 to 4; however, at day 5, virus levels collected from mice infected with ΔCTRL2 virus were slightly but significantly lower than in mice infected with CTRL2R (*P* ≤ 0.005) (Fig. 4A). To allow establishment of latent infection, we maintained infected mice for 28 days. For CTRL2R-infected mice, 90% of the mice survived to day 28 and generally did not succumb to infection before day 10. In contrast, for ΔCTRL2-infected mice, we observed slightly increased mortality beginning at day 7, with 15% fatality by day 10 and 19% fatality by day 28 (*P* ≤ 0.05 by log-rank test) (Fig. 4B). In general, acute viral replication and survival were largely similar for the mice infected with mutant or restored mutant viruses.

### Trigeminal ganglion infection with ΔCTRL2 and CTRL2R viruses.

**FIG 5:**
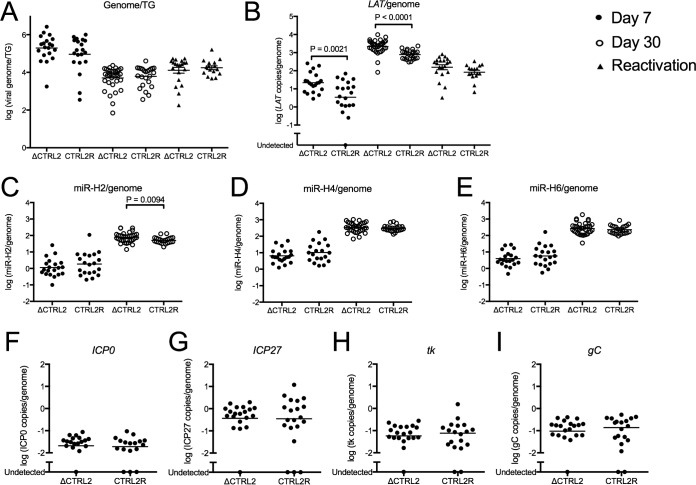
Viral DNA and transcript levels during acute and latent infection in mice infected with ΔCTRL2 or CTRL2R virus. TGs from infected mice were harvested at 7 dpi, 30 dpi, or following explant reactivation, and total RNA and DNA were isolated and quantified. (A) Viral genomes were measured by qPCR and normalized to the cellular DNA control. (B) Viral RNA transcript levels for *LAT* were measured at 7 and 30 dpi and following explant reactivation. (C to I) The miRNAs miR-H2 (C), miR-H4 (D), miR-H6 (E), and lytic transcripts *ICP0* (F), *ICP27* (G), *tk* (H), and *gC* (I) were measured by quantitative reverse transcription-PCR with specific primers and normalized to a cellular control and viral genome copy number. Statistical significance was evaluated with the Mann-Whitney test.

To test whether the ΔCTRL2 mutation affected the viral genome load in the ganglia during acute or latent infection, we isolated total ganglion DNA and quantified viral genomes at 7 and 30 dpi from TGs of mice infected with ΔCTRL2 or CTRL2R virus. Viral DNA levels, when normalized to cellular DNA levels, showed no significant difference in genomes per TG for ganglia acutely infected with ΔCTRL2 or CTRL2R viruses at day 7 or for ganglia latently infected with ΔCTRL2 or CTRL2R viruses at day 30 (Fig. 5A), showing that acute infection of the trigeminal ganglia was similar with the two viruses and there were similar levels of latent infection with the ΔCTRL2 and CTRL2R viruses.

To measure latent viral gene expression with the two viruses, total RNA was also extracted from each sample and measured with primers specific for the viral *LAT* intron, the viral miRNAs, including miR-H2, miR-H4, and miR-H6, the lytic viral transcripts, including *ICP0*, *ICP27* (IE), and the genes for thymidine kinase (*tk*; early kinetic class [E]), and glycoprotein C (*gC*; late kinetic class [L]), and also host *GAPDH* transcripts and *let-7a* miRNA. Viral transcripts were first normalized to cellular glyceraldehyde 3-phosphate dehydrogenase (*GAPDH*) mRNA. Total RNA levels were then normalized to total viral genome copy numbers for each mouse to calculate transcript levels per genome. Similarly, viral miRNAs were normalized to *let-7a* levels and then to the viral genome copy number for each mouse. In ganglia infected with the ΔCTRL2 virus, compared to those infected with the CTRL2R virus, we observed a small (~3-fold) but significant increase in levels of LATs at days 7 and 30 (Fig. 5B) (*P* ≤ 0.0001 by the Mann-Whitney test); however, the levels of *LAT* became indistinguishable during reactivation of ΔCTRL2 and CTRL2R viruses (Fig. 5B).

miR-H2 and miR-H4 are both derived from the primary *LAT* transcript (6, 17, 69); while miR-H6 is encoded upstream of the *LAT* transcription unit, its expression is dependent on a 200-bp sequence that includes the *LAT* promoter (6, 69). At day 7, miRNAs showed no significant difference; however, at day 30, we observed a small (~1.8-fold) increase in miR-H2 (Fig. 5C) in ΔCTRL2-infected ganglia, based on the Mann-Whitney test. Levels of miR-H4 (Fig. 5D) and miR-H6 (Fig. 5E) were not significantly different (~1.2-fold and ~1.4-fold higher, respectively). In addition, lytic transcript levels were not significantly different at day 7 (Fig. 5F to I). Because lytic transcript levels were too low for quantification at day 30, ganglia were scored as positive or negative for detectable transcripts. We did not find significant differences in the fraction of ganglia with detectable lytic viral transcripts between ΔCTRL2 and CTRL2R infections (Table 1). These results indicated that loss of CTCF binding at the *CTRL2* site increased the accumulation of LATs slightly but did not affect the low levels of lytic gene expression during latent infection; however, *LAT* levels became indistinguishable upon explant reactivation.

**TABLE 1:** Expression of viral lytic transcripts in mouse TG at 30 days

Virus	Expression of transcript (no. positive/total no.)			
	*ICP0*	*ICP27*	*tk*	*gC*
ΔCTRL2	8/34	8/34	7/34	7/34
CTRL2R	8/24	5/24	4/24	4/24

### Increased H3K27me3 histone modification on the *LAT* region of ΔCTRL2 mutant virus.

To examine the potential role of *CTRL2* in regulating viral chromatin during latency and its potential function as an insulator, we collected TGs from mice latently infected with ΔCTRL2 or CTRL2R viruses and performed ChIP analysis with antibodies specific for total histone H3 or heterochromatin modifications H3K9me3 and H3K27me3. The relative fraction of HSV-1 DNA immunoprecipitated was measured by qPCR using primers specific for the viral *LAT* promoter (*LAP*), *LAT* intron, and *ICP0* promoter normalized to the fraction of DNA immunoprecipitated by the cellular control, *GAPDH*.

**FIG 6:**
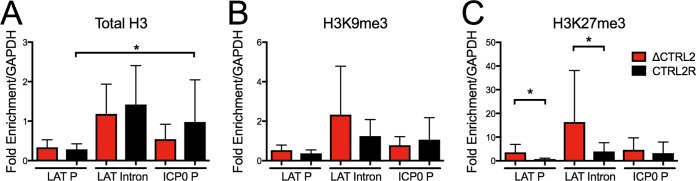
Deletion of *CTRL2* increased H3K27me3 accumulation on *LAT* promoter and intron sequences. Mice were infected with 2 × 10^5^ PFU/eye of ΔCTRL2 or CTRL2R virus. At 28 dpi, mice were sacrificed, TGs were harvested, and ChIP analysis was carried out with antibodies specific for total histone H3 (A), H3K9me3 (B), or H3K27me3 (C). Three viral regions, the *LAT* promoter (*LAP*), *LAT* intron, and *ICP0* promoter (ICP0 P), were examined, and the results are expressed as means and standard deviations of the percent viral chromatin immunoprecipitated relative to immunoprecipitation of the cellular *GAPDH* region. Asterisks indicate significance (*P* < 0.05), which was evaluated using the Wilcoxon matched-pairs signed-rank test.

Similar to previous results with wild-type (WT) KOS and 17syn^+^ HSV-1 strains (22, 23), the CTRL2R virus *LAP* sequences were associated with less silenced chromatin than the *LAT* intron or *ICP0* promoter regions during latent infection (Fig. 6A and B). We observed ~3- to 5-fold less total H3, H3K9me3, and H3K27me3 associated at *LAP* relative to that at the *ICP0* promoter (*P* ≤ 0.05, Wilcoxon matched-pairs signed-rank test). In contrast, during latent infection with the ΔCTRL2 mutant virus, the *LAP* sequences and *ICP0* promoter region were associated with equivalent levels of H3 histones and H3-directed heterochromatin modifications. Specifically, we observed a mean fold enrichment of less than 1.5-fold at the *ICP0* promoter relative to that of the *LAP*s for all antibodies tested, and this did not reach statistical significance. These results showed that CTCF binding at the *CTRL2* region serves as a chromatin barrier to limit accumulation of H3K27me3 at the *LAP* and the *LAT* intron.

When we compared the chromatin of the ΔCTRL2 virus directly to that of the CTRL2R virus, we observed a significant increase of H3K27me3 heterochromatin marker accumulation at *LAP* and the *LAT* intron (*P* ≤ 0.05), but not at the *ICP0* promoter (Fig. 6C). We also observed slight but not significant increases in total histone H3 or H3K9me3 accumulation. These results showed that CTCF binding to *CTRL2* may prevent the spread of specific heterochromatin markers, such as H3K27me3, to the *LAT* region encompassing *LAP* and the *LAT* intron.

### Explant reactivation from TGs infected with ΔCTRL2 virus is reduced.

**FIG 7:**
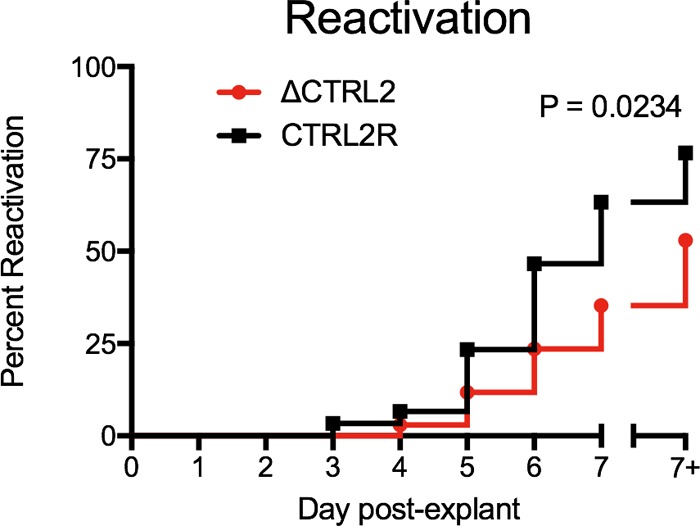
Explant reactivation was reduced by infection with ΔCTRL2 mutant virus. Mice were infected with 2 × 10^5^ PFU/eye of ΔCTRL2 or CTRL2R virus. At 28 dpi, individual ganglia from each mouse, from 15 mice from two independent infections, were isolated and explanted onto Vero cell monolayers. Supernatant was collected daily from the overlay medium for 7 days. At 7 days, the underlying cells were also collected (7+). All samples were replated onto fresh Vero monolayers to detect infectious virus. Statistical significance was evaluated with the log-rank Mantel-Cox test.

To determine whether loss of CTCF binding at the *CTRL2* sites affected the ability of the ΔCTRL2 mutant virus to reactivate, we harvested TGs latently infected with ΔCTRL2 or CTRL2R virus and explanted them onto a monolayer of Vero cells. We tested individual ganglia from two independent infections of 5 mice and 10 mice per virus, respectively. We assessed the emergence of infectious virus for 7 days postexplant by collecting overlay medium and, on day 7, also the underlying Vero cell monolayer and replating each on a fresh Vero monolayer. Infectious virus was first detected at 3 days from CTRL2R virus-infected ganglia but appeared first at 4 days from ΔCTRL2 virus-infected ganglia (Fig. 7). By 7 days postexplant, 77% of CTRL2R virus-infected ganglia produced infectious virus, while only 53% of ΔCTRL2 virus-infected ganglia produced detectable virus (*P* ≤ 0.03, by log-rank test). At 7 days, the underlying cells were also sampled, and ΔCTRL2 never reached the reactivation frequency of the CTRL2R virus, as evaluated by the log-rank Mantel-Cox test. These results indicated that despite similar numbers of latent viral genomes present at day 28, genomes from ΔCTRL2 virus infection showed significantly reduced explant reactivation.

## DISCUSSION

The *LAT* and *ICP0* transcriptional units are encoded on opposite strands of the HSV-1 genome (Fig. 1) but are regulated independently during latent infection, so that they maintain suppression of *ICP0* gene transcription while allowing persistence of *LAT* transcription (4). The divergent expression patterns correlate with different chromatin modifications found at the *LAT* and *ICP0* promoters that are maintained despite the relative proximity of these genetic elements (21–23, 70, 71). While *ICP0* and other lytic gene promoters are associated with high levels of heterochromatin during latent infection, the *LAT* region also shows enrichment of euchromatin (23, 70). CTCF is a key mediator of the ability to maintain independently regulated but adjacent chromatin domains within mammalian and viral genomes (51, 72). Of the various sites on the HSV-1 genome that bind CTCF during latent infection, *CTRL2* has been of particular interest due to its position between the transcriptionally active *LAT* promoter and the silenced *ICP0* promoter (64–66).

In this study, we constructed a mutant HSV-1 virus, ΔCTRL2, with deletions of the *CTRL2* CTCF-binding sites to examine the role of CTCF binding to *CTRL2* during *in vivo* latent infection in a mouse model system. The ΔCTRL2 mutant virus produced a relatively normal acute infection in the cornea and trigeminal ganglia and established latent infection to the same level as the CTRL2R virus. The ΔCTRL2 mutant virus showed slightly higher levels of *LAT* expression during latent infection, but this was indistinguishable during reactivation. Despite equal latent DNA genome loads per TG, the mutant virus showed reduced reactivation relative to that by the CTRL2R virus. Because the mutant virus showed higher levels of H3K27me3 heterochromatin on the *LAT* promoter and intron sequences, we hypothesize that CTCF binding at the *CTRL2* site serves as a chromatin barrier to keep at least certain forms of heterochromatin off the *LAT* promoter/regulatory sequences.

### CTCF is associated with the *LAT* intron sequences in a *CTRL2*-dependent manner.

We confirmed that deletion of the *CTRL2* region was sufficient to reduce CTCF binding to the *LAT* intron to background levels. CTCF is known to mediate long-range, three-dimensional chromatin interactions through simultaneous association with distant binding sites; therefore, the loss of one binding site has the potential to alter CTCF binding to distant sites (54, 73–75). Our study also identified a reduction of CTCF binding at the *ICP0* promoter region after deletion of the *CTRL2* sites, suggesting that *CTRL2* and a region near the *ICP0* promoter may bind CTCF cooperatively. An interaction between these sites could form a closed chromatin loop surrounding the 5′ end of the *ICP0* gene and separating the *ICP0* promoter from the *LAT* promoter. However, there are CTCF-binding sites at many regions of the HSV-1 genome, and further work is required to characterize these sites, their role in the HSV-1 3D chromatin architecture, and their interaction with the *LAT* region.

### Removal of CTCF binding to *CTRL2* decreases viral reactivation in a mouse model.

Deletion of the CTCF binding site at the *CTRL2* region did not affect viral replication in lytic infection of cultured HeLa or HFF cells or acute infection at the ocular epithelium for the first 4 days of infection. These results argue that lytic infection of nonneuronal cells is not affected by CTCF binding to *CTRL2*. This is consistent with the findings of a recent study that did not detect CTCF binding at the *CTRL2* site during lytic infection (68). Although infection with the ΔCTRL2 virus showed a reduction in shed virus in the eyes of mice on one day, at 5 dpi, total viral genomes per TG were not significantly different between ΔCTRL2 and CTRL2R viruses at 7 or 30 dpi. However, the mutant virus showed reduced reactivation upon ganglionic explant, arguing that CTCF binding at *CTRL2* promotes reactivation. Slightly higher levels of *LAT* were observed during latent infection with the mutant virus compared to the repaired virus, but during reactivation, the levels of *LAT* were equivalent. Therefore, the major effect of the *CTRL2* sites on viral infection seems to be promotion of reactivation.

Results from related herpesviruses, EBV and KSHV, have also indicated that CTCF has a complex role in promoting latent infection. Removal of CTCF-binding sites from the intron of the *LMP2A* gene of EBV resulted in a higher viral genome copy number in latent infection, possibly as a result of partial lytic replication or aberrant latent replication (76). However, results with KSHV indicated that removal of CTCF-binding sites from the intron of its major latency-associated transcript reduced the latent genome copy number due to reduced viral episome maintenance (61). Given that CTCF binding has such diverse effects among related herpesviruses, it is possible that CTCF binding exhibits both positive and negative regulation during HSV-1 latent infection. To address these questions, future studies that address the progression of latency establishment before 28 days and the maintenance of long-term latent viral genomes are needed to differentiate between the initial viral dose entering the ganglia, viral replication within the neural tissue, and long-term viral genome maintenance.

### Histone modifications at the *LAT* promoter sequences are affected by CTCF binding at the *CTRL2* site.

The increased accumulation of H3K27me3-modified heterochromatin at *LAP* and the *LAT* enhancer regions of the ΔCTRL2 mutant viral genome is consistent with the hypothesis that CTCF binding at *CTRL2* functions as an insulator to block the linear spread of heterochromatin from the lytic *ICP0* region to the *LAT* transcriptional regulatory regions. Previous results demonstrated that the H3K27me3 modification accumulates gradually at viral lytic promoters from days 10 to 14 during the establishment of latent infection and is enhanced by LATs (20, 23). Therefore, increased *LAT* accumulation at 7 dpi with the ΔCTRL2 virus may also contribute to higher levels of H3K27me3 accumulation at the *LAT* sequences by day 28.

Furthermore, H3K27me3 can be found in bivalent chromatin domains (77) and poised chromatin domains (78) on developmentally regulated genes and genes in pluripotent stem cells. Our previous study found that increased levels of H3K27me3 at the *ICP8* promoter relative to the viral *U_L_48* promoter correlated with higher levels of *ICP8* RNA than *U_L_48* RNA (20). Therefore, H3K27me3 alone may be insufficient to silence transcription, or additional histone modifications, such as H3K4me3, may exert a dominant effect to maintain active transcription. Alternatively, a phospho switch proposed for reactivation (79) may work effectively on chromatin that has H3K27me3 modifications. Therefore, H3K27me3 may be important for maintaining repressed but poised genomes during latent infection.

### Model for mechanism of action for CTCF binding at the *CTRL2* site.

We have shown that binding of CTCF to the *CTRL2* sites causes a statistically significant reduction in H3K27me3 and a trend toward reduction of H3K9me3 modification of chromatin on the *LAT* promoter and intron sequences. Furthermore, the *CTRL2* sites promote the reactivation of latent virus upon explant in culture. We propose a model in which CTCF bound at *CTRL2* dimerizes with a CTCF molecule bound at another site to form a 3D structure that promotes reactivation. The most likely targets, VP16 and ICP0, are viral genes whose products are involved in early stages of reactivation (1, 12, 80). We envision at least two ways in which CTCF promotes their expression during reactivation. First, we have shown that CTCF binding at the *CTRL2* sites promotes CTCF binding in the *ICP0* promoter, which could place the 5′ end of the *ICP0* gene in a chromatin loop. Because CTCF-mediated chromatin loops are believed to often enclose inducible gene regulatory domains (81), enclosing the *ICP0* gene promoter in a chromatin loop may promote its expression when reactivation is triggered. Second, CTCF may dimerize with a CTCF molecule bound near the *VP16*/*UL48* gene promoter to keep the VP16 promoter in a chromatin state that can be readily induced when reactivation is triggered. Further studies are needed to define the 3D chromatin structure of viral chromatin that is dependent on CTCF binding at the *CTRL2* sites and to test these hypotheses.

### Establishing and maintaining a poised latent infection.

To establish a successful latent infection, HSV-1 must balance suppression of lytic gene expression with maintenance of a latent state that is poised for reactivation. The results in this study indicate that HSV-1 exploits the cellular protein CTCF to maintain its genome in a state that can be efficiently reactivated. Large domains of H3K27me3 with short domains of H3K4me3 histone modifications are known to define a bivalent chromatin structure that is silenced but poised to be reactivated at the appropriate time (77). We propose a model to explain how the *CTRL2* sites can promote reactivation. In addition to the *CTRL2* sites, HSV-1 gene products contribute to promoting H3K27me3 on HSV-1 lytic gene chromatin. HSV-1 *LAT* promotes H3K27me3 on lytic gene chromatin (23) as well as reactivation (12; P. Raja, J. S. Lee, and D. M. Knipe, unpublished results). Furthermore, ICP0 promotes *LAT* expression and H3K27me3 modifications on lytic gene chromatin (44) as well as reactivation (82, 83). Therefore, CTCF, *LAT*, and ICP0 may all be acting to promote the correct form of viral chromatin so that a poised latent infection is established and maintained and reactivation can take place at the appropriate time. Therefore, viral gene products play an active role in not only promoting epigenetic silencing of DNA viral genomes for latent infection (84) but also keeping them in a poised form that is capable of efficient reactivation, a state that we call poised latency.

## MATERIALS AND METHODS

### Cells and viruses.

Vero, HeLa, and HFF cells were obtained from ATCC. The ΔCTRL2 virus has a deletion of *CTRL2* sites from within the *LAT* intron of both TR_L_ repeats (HSV-1 KOS KT899744 bp 120136 to 120508 [85]).

The control CTRL2R virus was constructed in parallel by cotransfection of the WT DNA fragment. All viruses were propagated and titrated in parallel on Vero cells.

### Mouse infections.

Mice were housed in accordance with institutional and National Institutes of Health guidelines on the care and use of animals in research. Ocular infection was carried out with 2 × 10^5^ PFU/eye of virus (86). Eyes were swabbed on days 1 to 5 postinfection, and virus collected from tear films was titrated on Vero cells (87). Mice were monitored for survival for at least 28 dpi.

### ChIP assays.

Immunoprecipitations were carried out on chromatin prepared from TGs (20) with 5 to 10 μg of anti-CTCF (catalog number 07-729; Millipore), 2.5 μg of anti-histone H3 (ab1791; Abcam, Inc.), 2.5 μg anti-H3K27me3 (39156; Active Motif, Inc.), 2.5 μg of anti-H3K9me3 (ab8580; Abcam, Inc.), or normal rabbit IgG (12-370; Millipore). Immunoprecipitated DNA was quantitated using the qPCR primers listed in Table 2.

**TABLE 2:** Primer sequences

**Target**	**Purpose**	**Sequence (5′**–**3′)**	
		**Forward**	**Reverse**
*LAT* promoter	qPCR	5′-CCCGGCCCGCACGAT-3′	5′-CAACACCCCGCCGCTTT-3′
*LAT* intron	qPCR	5′-GGGTCATCCAGAGGCTGTTC-3′	5′-GTGGACCAGACGGGAAACAT-3′
*ICP0* promoter	qPCR	5′-CGCCTTCCCGAAGAAACTCA-3′	5′-CGCTCAATGAACCCGCATT-3′
*ICP8* promoter	qPCR	5′-GAGACCGGGGTTGGGGAATG AATC-3′	5′-CCCCGGGGGTTGTCTGTGAAG G-3′
*GAPDH* pseudogene	qPCR	5′-TTCGACAGTCAGCCGCATCTTCTT-3′	5′-CAGGCGCCCAATACGACCAA ATC-3′

### Quantification of viral genomes and transcripts.

Nucleic acids were isolated using the Allprep RNA/DNA minikit (Qiagen), and RNA was reverse-transcribed with specific primers using the QuantiTect RT kit (Qiagen). Viral DNA and transcripts were quantified relative to host *adipsin* DNA and *GAPDH* mRNA (44, 88). For miRNA quantification, RNAs from TGs were purified using an RNeasyPlus minikit (Qiagen) and quantified using TaqMan miRNA assays (Applied Biosystems). Viral miRNA levels were normalized to cellular *let-7a*.

### Reactivation.

TGs isolated from mice latently infected with HSV-1 ΔCTRL2 or CTRL2R viruses were bisected and explanted onto a confluent monolayer of Vero cells in Dulbecco’s modified Eagle’s medium supplemented with 10% (vol/vol) fetal bovine serum and 0.25 μg/ml amphotericin B. Culture medium was sampled daily for 7 days, and after 7 days (7+) the entire Vero monolayer and ganglia were collected, frozen, and replated onto a fresh Vero monolayer to score the number of ganglia that showed detectable infectious virus (see Text S1 for additional details on our methods).

10.1128/mBio.02372-17.1 TEXT S1  Supplemental methods. Download TEXT S1, DOCX file, 0.1 MB. Copyright © 2018 Lee et al. 2018 Lee et al. This content is distributed under the terms of the Creative Commons Attribution 4.0 International license.
