# Supplementary material for: CCCTC-Binding Factor Acts as a Heterochromatin Barrier on Herpes Simplex Viral Latent Chromatin and Contributes to Poised Latent Infection
Source: mBio. 2018 Feb 6;9(1):e02372-17. doi: 10.1128/mBio.02372-17 (PMC5801469; doi:10.1128/mBio.02372-17)
Supplement: TEXT S1 [file mbo001183705s1.docx]

**SUPPLEMENTAL MATERIAL S1**

**MATERIALS AND METHODS**

**Cells and viruses**

Vero, HeLa, and primary human foreskin fibroblast (HFF) cells were obtained from the American Type Culture Collection (Manassas, VA) and maintained in Dulbecco’s modified Eagle medium (DMEM) and supplemented with 10% (v/v) fetal bovine serum (FBS), 2 mM L-glutamine at 37°C.

Viruses used in this study were constructed in the HSV-1 KOS wildtype (WT) genetic background (1). We first generated a *LAT* deletion mutant virus, KOSΔLAT1.8eGFP, with an eGFP cassette inserted in the *LAT* coding sequences, and then used that virus to generate the ΔCTRL2 *LAT* deletion mutant virus and the CTRL2R *LAT* restored virus. To generate the pΔLAT1.8eGFP plasmid, the *eGFP* expression cassette from the *Ase*I to *Mlu*I fragment of the pEGFP.C2 plasmid (Clontech) was inserted into the *Pst*I site of the pΔLAT1.8 plasmid (2). The pΔLAT1.8eGFP plasmid was cotransfected with infectious WT HSV-1 KOS strain DNA into Vero cells, and recombinant viruses were plaque-purified and screened for GFP expression to generate KOSΔLAT1.8eGFP virus. The pBS.LAT.flank plasmid was constructed by PCR amplification of ~4.6 kbp from WT KOS DNA in the region of the *LAT* intron and ~1.5 kbp of flanking sequences and insertion into the *EcoRI* site of pBluescript II SK+ vector.

The ΔCTRL2 mutation was constructed using site-directed mutagenesis to introduce a *BamH*I site into pBS.LAT.flank (Figure 1A). Subsequent *Hpa*I and *Bam*HI digestions yielded a plasmid pLATΔCTRL2 with a 370 bp deletion across the *CTRL2* site. Linearized pLATΔCTRL2 plasmid and KOSΔLAT1.8eGFP infectious viral DNA were co-transfected into Vero cells to generate the ΔCTRL2 mutant virus via homologous recombination. Progeny viruses were screened for loss of GFP expression, plaque-purified three times, and confirmed with diagnostic PCR and sequencing across the *LAT* promoter-intron region. The ΔCTRL2 virus has a deletion of CTCF binding sites at *CTRL2* from within the *LAT* 2.0 kbp intron of both TR_L_ repeats (HSV-1 KOS KT899744 bp 120136-120508) without disrupting the nearby *LAT* intron splice sites, branch points, or the *ICP0* polyadenylation signal sequence.

The control CTRL2R virus was constructed in parallel by co-transfection of the WT DNA fragment from pBS.LAT.flank. All viruses were propagated and titrated in parallel on Vero cells. To assess lytic replication, HFF or HeLa cells were infected at 3 PFU/cell for 24 h. Cells were scraped in medium, frozen, and sonicated, and viral yields were titrated on Vero cells.

**Mouse infections**

Mice were housed in accordance with institutional and National Institutes of Health guidelines on the care and use of animals in research. The Institutional Animal Care and Use Committee of Harvard Medical School approved all procedures. Six-week-old CD1 mice (Charles River Laboratories) were anesthetized by intraperitoneal injection of ketamine hydrochloride (3.4 mg/mouse) and xylazine hydrochloride (0.5 mg/mouse). Mice were then inoculated with 2 x 10^5^ PFU/eye of virus in a 7.5 μl volume onto scarified corneas as described previously (3). Eyes were swabbed on days 1 to 5 post infection (dpi), and virus collected from tear films was titrated on Vero cells as described previously (4). Mice were monitored for survival for at least 28 dpi.

**Chromatin immunoprecipitation (ChIP) assays**

Chromatin was prepared from trigeminal ganglia (TGs) as described previously (5) with modifications. Intact TGs were fixed in 1% formaldehyde for 15 min at 37°C. Formaldehyde was quenched with 0.125 M glycine for 3 min at room temperature. TGs were washed 3x with cold PBS and then homogenized in PBS with the TissueLyser LT and 5 mm stainless steel beads (Qiagen) at 50 oscillations/sec for 6 min. Subsequently the homogenate was dissolved in SDS lysis buffer (1% SDS, 10 mM EDTA, 50 mM Tris, pH 8.1) and sonicated to yield chromatin fragments of approximately 500 bp or smaller using a Biorupter 200 (Diagenode) for 6 cycles of 5 min each (15 sec ON, 45 sec OFF) at a high power setting. Immunoprecipitation (IP) reactions contained 50 μg of chromatin diluted 10-fold in ChIP dilution buffer (150 mM NaCl, 10 mM Na_2_HPO_4_, 2 mM EDTA, 1.1% Triton, 0.1% SDS). From each immunoprecipitation reaction, 1% of the chromatin was removed and reserved for input measurements. Immunocomplexes were formed by overnight incubation at 4°C with antibody as follows: 5-10 μg of anti-CTCF (Millipore, 07-729), 2.5 μg of anti-histone H3 (Abcam, ab1791), 2.5 μg anti-H3K27me3 (Active motif, 39156), 2.5 μg of anti-H3K9me3 (Abcam, ab8580), or normal rabbit IgG (Millipore, 12-370) as a negative control. Antibody complexes were captured with 20 μl Magna ChIP protein A magnetic beads (Millipore) by incubation with IP samples at 4°C for 2 h.

Antibody complexes were washed 3x with ChIP dilution buffer containing 0.1% SDS and 1 mM PMSF, 3x with lithium chloride wash buffer (50 mM HEPES, pH 7.5, 500 mM LiCl, 1 mM EDTA, 1% NP-40, 0.7% sodium deoxycholate, 1 mM PMSF), and once with Tris-EDTA pH 8.0 buffer. Complexes were eluted from beads twice with the addition of 90 μl of elution buffer (1% SDS, 0.1 M NaHCO_3_) for 10 min at 65°C. Formaldehyde cross-linking was reversed by addition of NaCl to a concentration of 200 mM and incubation for 30 min at 95°C, and the DNA was purified and isolated by treatment with 1 μg of RNase A (Ambion) at 37°C for 1 h, proteinase K at 45°C for 2 h, and use of the QIAquick PCR Purification kit (Qiagen) according to the manufacturer’s instructions.

**Quantification of DNA from ChIP using quantitative PCR**

Quantitative PCR (qPCR) was performed as described previously (6) using Power SYBR green master mix and a Prism 7300 sequence detection system (Applied Biosystems) according to the manufacturer’s directions. Reactions were performed in a total volume of 25 μl, with 2.5 μl of DNA, and a final concentration of 100 nM of each qPCR primer, listed in Table 1. The specificity of each primer pair was confirmed by running dissociation curves of the PCR products for each reaction. Samples were run in duplicate, and relative copy numbers were determined by comparison to a standard curve generated by 10-fold serial dilution of DNA isolated from HSV-1 infected HFF cells. The fraction of viral DNA immunoprecipitated from each reaction was compared to the input sample, and presented as percent immunoprecipitated for CTCF, and as fold-enrichment relative to cellular control for histone H3, H3K27me3, and H3K9me3 by normalization to the fraction of cellular *glyceraldehyde 3-phospate dehydrogenase* (*GAPDH*) gene sequences precipitated from the same reaction. ChIP assays were carried out from at least 3 independent experiments from at least 3 independent infections with ΔCTRL2 or CTRL2R.

**RNA and DNA isolation and quantification of viral transcripts**

Quantification of viral transcripts from trigeminal ganglia of HSV-1 infected mice was performed as previously described (7, 8). Briefly, RNA and DNA were isolated using the Allprep RNA/DNA minikit (Qiagen), and reverse transcription of RNA was performed using the QuantiTect RT kit (Qiagen) according to the manufacturer’s instructions, except using specific primers as described previously (8). Serially diluted standards as described previously (5, 8-10) were used to quantify viral DNA and transcripts relative to host *adipsin* DNA and *GAPDH* mRNA. Standard curves were linear in all cases (with R^2^ values over 0.99). The detection limit of the *ICP0*, *ICP27*, *tk*, and *gC* assays was 100 copies/TG. For miRNA quantification, total RNAs from TGs were purified using RNeasyPlus Mini Kit (Qiagen) following the protocol for small RNA purification provided by the manufacturer. miRNA levels were assessed using TaqMan miRNA assays (Applied Biosystems) according to the manufacturer’s instructions. Viral miRNA levels were normalized to cellular *let-7a* levels. Differences in quantified transcripts per genome were analyzed statistically using the Mann-Whitney test. Data in Table 2 represent the presence of detectable lytic RNAs per ganglion, as confirmed by the presence of transcript-specific peaks in qPCR melt curves that were absent in mock-infected samples tested. The fraction of RNA-positive ganglia was analyzed statistically by Fisher’s exact test.

**Reactivation of HSV-1 from latently infected mouse TGs**

Individual TGs were isolated from mice infected with HSV-1 ΔCTRL2 or CTRL2R viruses at least 28 days prior to collection. TGs were bisected and explanted on a confluent monolayer of Vero cells in DMEM supplemented with 10% (v/v) FBS and 0.25 μg/ml amphotericin B, in a 6-well plate at 37°C, containing one ganglion per well. At 24 h intervals for 7 days, 100 μl of the culture overlay media was removed and re-plated onto a fresh Vero monolayer to detect infectious virus. After 7 days the entire Vero monolayer and ganglia were collected, frozen, and replated onto a fresh Vero monolayer to score the number of ganglia that showed detectable infectious virus.

**REFERENCES**

1. **Colgrove RC, Liu X, Griffiths A, Raja P, Deluca NA, Newman RM, Coen DM, Knipe DM.** 2016. History and genomic sequence analysis of the herpes simplex virus 1 KOS and KOS1.1 sub-strains. Virology **487:**215-221.

2. **Leib DA, Bogard CL, Kosz-Vnenchak M, Hicks KA, Coen DM, Knipe DM, Schaffer PA.** 1989. A deletion mutant of the latency-associated transcript of herpes simplex virus type 1 reactivates from the latent state with reduced frequency. J Virol **63:**2893-2900.

3. **Tenser RB, Dunstan ME.** 1979. Herpes simplex virus thymidine kinase expression in infection of the trigeminal ganglion. Virology **99:**417-422.

4. **Coen DM, Irmiere AF, Jacobson JG, Kerns KM.** 1989. Low levels of herpes simplex virus thymidine- thymidylate kinase are not limiting for sensitivity to certain antiviral drugs or for latency in a mouse model. Virology **168:**221-231.

5. **Cliffe AR, Coen DM, Knipe DM.** 2013. Kinetics of facultative heterochromatin and polycomb group protein association with the herpes simplex viral genome during establishment of latent infection. MBio **4:**e00590-00512.

6. **Cliffe AR, Knipe DM.** 2008. Herpes simplex virus ICP0 promotes both histone removal and acetylation on viral DNA during lytic infection. J Virol **82:**12030-12038.

7. **Raja P, Lee JS, Pan D, Pesola JM, Coen DM, Knipe DM.** 2016. A Herpesviral Lytic Protein Regulates the Structure of Latent Viral Chromatin. MBio **7:**e00633-00616.

8. **Pan D, Flores O, Umbach JL, Pesola JM, Bentley P, Rosato PC, Leib DA, Cullen BR, Coen DM.** 2014. A neuron-specific host microRNA targets herpes simplex virus-1 ICP0 expression and promotes latency. Cell Host Microbe **15:**446-456.

9. **Cliffe AR, Garber DA, Knipe DM.** 2009. Transcription of the herpes simplex virus latency-associated transcript promotes the formation of facultative heterochromatin on lytic promoters. J Virol **83:**8182-8190.

10. **Orzalli MH, Conwell SE, Berrios C, Decaprio JA, Knipe DM.** 2013. Nuclear interferon-inducible protein 16 promotes silencing of herpesviral and transfected DNA. Proc Natl Acad Sci U S A **110:**E4492-4501.
